# Potential Links between Impaired One-Carbon Metabolism Due to Polymorphisms, Inadequate B-Vitamin Status, and the Development of Alzheimer’s Disease

**DOI:** 10.3390/nu8120803

**Published:** 2016-12-10

**Authors:** Barbara Troesch, Peter Weber, M. Hasan Mohajeri

**Affiliations:** DSM Nutritional Products Ltd., Wurmisweg 576, Kaiseraugst 4303, Switzerland; peter.weber@dsm.com (P.W.); hasan.mohajeri@dsm.com (M.H.M.)

**Keywords:** homocysteine, dementia, Alzheimer’s disease, nutrition, one-carbon metabolism, B-vitamins, polymorphism, prevention, therapy

## Abstract

Alzheimer’s disease (AD) is the major cause of dementia and no preventive or effective treatment has been established to date. The etiology of AD is poorly understood, but genetic and environmental factors seem to play a role in its onset and progression. In particular, factors affecting the one-carbon metabolism (OCM) are thought to be important and elevated homocysteine (Hcy) levels, indicating impaired OCM, have been associated with AD. We aimed at evaluating the role of polymorphisms of key OCM enzymes in the etiology of AD, particularly when intakes of relevant B-vitamins are inadequate. Our review indicates that a range of compensatory mechanisms exist to maintain a metabolic balance. However, these become overwhelmed if the activity of more than one enzyme is reduced due to genetic factors or insufficient folate, riboflavin, vitamin B6 and/or vitamin B12 levels. Consequences include increased Hcy levels and reduced capacity to synthetize, methylate and repair DNA, and/or modulated neurotransmission. This seems to favor the development of hallmarks of AD particularly when combined with increased oxidative stress e.g., in apolipoprotein E (ApoE) ε4 carriers. However, as these effects can be compensated at least partially by adequate intakes of B-vitamins, achieving optimal B-vitamin status for the general population should be a public health priority.

## 1. Introduction

Alzheimer’s disease (AD) is the most frequent type of dementia, causing around two-thirds of cases [1]. The condition becomes more common with increasing age, affecting between 5% and 8%, 15% and 20%, and 25% and 50% of those in the age groups ≥65 years, ≥75 years and ≥85 years, respectively [1]. The number of people aged ≥65 years is estimated to increase from ~500 million in 2008 to ~1.3 billion in 2040 [2]. A significant increase in absolute numbers, but also in the proportion of the population affected by the disease, is expected for the coming decades. In Europe, the predicted increase in numbers of individuals with dementia from ~36 million in 2010 to ~115 million in 2050 will result in an around €250 billion health care cost with respect to the condition by 2030 [1]. In the U.S., the cost is projected to exceed $1 trillion by 2050 if the disease continues to progress at its current pace [3]. Even more importantly, despite intensive research, there is currently no treatment available to cure or reverse AD [4]. This is reflected in the alarming mortality rates: for diseases such as human immunodeficiency virus, cardiovascular disease (CVD) and some cancers, important decreases in the death rate were achieved between 2000 and 2010, while for AD, the death rate increased by nearly 70% during the same period [5].

While many questions remain concerning its etiology, treatment is further complicated by the early onset of neuro-pathological changes: postmortem studies have revealed specific hallmarks of AD such as amyloid plaque formations in cognitively normal elderly [6]. It has been postulated that they develop decades before even mild symptoms of dementia manifest [7]. By the time the disease is diagnosed, cellular damage and amyloid plaque deposition might therefore already be too advanced for treatment to be successful [8,9]. Consequently, preventing the development o AD seems to be a promising avenue for improving health and quality of life for the elderly and to reduce the burden for society.

For a preventive approach to be successful, a better understanding of risk factors for AD is crucial. Some rare genetic mutations have been identified as the cause of early onset of the disease [10], but only a relatively small fraction of cases falls into this category. We will therefore concentrate on the significantly more common late-onset type, which is thought to be triggered by a combination of genetic, epigenetic and environmental factors [11]. It has been well established that apolipoprotein E (ApoE) is a very important genetic risk factor for age-dependent chronic diseases, including CVD and AD [12]. Due to two major polymorphisms on the encoding exon 4 of this gene, three major protein isoforms, ApoE ε2, ApoE ε3 and ApoE ε4 exist [13]. It has been shown that homozygous carriers of the ApoE ε4 allele have a more than 10-fold increased risk of developing AD, possibly due to increased cholesterol levels, altered brain development early in life [12] or increased oxidative brain damage [14]. 

Environmental factors such as nutrition seem to play a role in the development of the disease [11]. In particular, some B-vitamins are thought to be implicated, even though the mechanism linking low status of B-vitamins and the development of AD is poorly understood. However, it seems that elevated levels of homocysteine (Hcy), a non-protein sulfur-containing amino acid implicated in the etiology of a range of medical conditions such as CVD [15], play an important role. Insufficiency of B-vitamins may also affect the development of the diseases via their role in DNA methylation [16], synthesis and/or repair [17] or in modulating neurotransmission [18]. Polymorphisms in genes encoding for specific enzymes can significantly affect their activity [19]. Therefore, studying mutations at critical steps in the metabolism of B-vitamins might help resolve some of the inconsistencies reported for their protective effect on the development of AD. Our aim is to evaluate the role of common polymorphisms of key enzymes in one-carbon metabolism (OCM; See Table 1) in the etiology of AD, particularly when intakes of the relevant B-vitamins are inadequate.

## 2. Evidence Linking B-Vitamins, Hcy and the Pathogenesis of AD

### 2.1. Observational Trials

Epidemiological studies provide evidence that AD patients tend to have higher Hcy plasma levels than controls, while there are trends for lower levels of B-vitamins [9,32]. Follow-up of a cohort with initially dementia-free elderly for a median of eight years found that plasma Hcy level >14 µmol/L at baseline doubled the risk of developing AD [33]. In addition, the inverse association between Hcy levels and cognitive decline seemed to exist even when the former was in what is generally accepted as the normal range (≤15 µmol/ L) [34]. A meta-analysis in 2011 concluded that Hcy levels were clearly higher in AD patients compared to controls [32]. However, based on the available evidence, they could not establish that hyperhomocysteinemia preceded AD [32]. McCaddon and Miller [35] concluded that the available evidence showed a strong and coherent relationship, biological plausibility, dose-response relationship and temporality, and therefore, most of the criteria necessary to establish causality between elevated Hcy and dementia were fulfilled. In the studies they reviewed, elevated Hcy predated hallmarks of AD such as dementia, brain atrophy or neurofibrillary tangles by 5–35 years [35]. They felt what was needed were well-designed intervention trials showing a clear effect of Hcy lowering on cognitive decline [35] (see below). A more recent meta-analysis concluded that the relative risk of developing AD due to elevated Hcy levels or low folate levels were ~1.8 (95% confidence interval 1.37–2.16) and ~2.1 (95% confidence interval 1.51–2.71), respectively, while the data for vitamin B12 and AD was inconclusive, even though AD patients had lower serum levels than controls [36]. 

### 2.2. Evidence from Supplementation with B-Vitamins

While somewhat inconsistent, some of the studies supplementing B vitamins show promising results: In a randomized placebo-controlled trial in elderly men, supplementation with 2 mg folic acid, 25 mg vitamin B6 and 400 µg vitamin B12 daily for 2 years decreased Hcy levels and reduced the rate of increase in circulating levels of amyloid-β1-40, an indicator for AD [37]. Supplementation with 800 µg folic acid daily for 3 years also led to a reduced progression of cognitive decline in parallel with a decrease in Hcy plasma levels compared to a control group receiving a placebo [38]. Moreover, an intervention with B-vitamins (800 µg folic acid, 500 µg vitamin B12 and 20 mg vitamin B6 per day for 2 years) in elderly with mild cognitive impairment was shown to slow down the progression of brain atrophy and reduce Hcy levels, both of which were associated with improved cognitive performance [39]. More specifically, this intervention decelerated shrinkage of the grey matter regions of the brain that are particularly affected by AD and the protective effect of the B-vitamins was limited to those with elevated Hcy levels [40]. Doses of these vitamins well above the recommended daily intakes in elderly men (aged ≥75 years) who were not specifically selected for elevated Hcy levels led to an improvement in vitamin status and Hcy levels [37]. In addition, these doses slowed the increase in circulating levels of amyloid beta, a proposed indicator for amyloid plaque formation, even though it did not reach statistical significance [37]. An intervention with supplements in a similar range improved memory and reduced the rate of atrophy in regions particularly affected by AD in elderly with mild cognitive impairment, particularly if they had elevated Hcy levels [39,40]. 

However, despite some encouraging results, a study on the benefits of Hcy lowering on heart health concluded that the evidence did not support the recommendation of routine supplementation with B-vitamins [41]. Similarly, despite lowering Hcy by around 25%, B-vitamin supplementation only had a marginal effect on cognitive aging [42]. McCaddon and Miller [35] pointed out that most individuals included did not actually experience such a decline and they highlighted the need for further studies specifically designed to assess such an effect.

## 3. Role of Key Polymorphisms in the OCM

### 3.1. Overview of the Enzymes of the OCM

**Figure 1:**
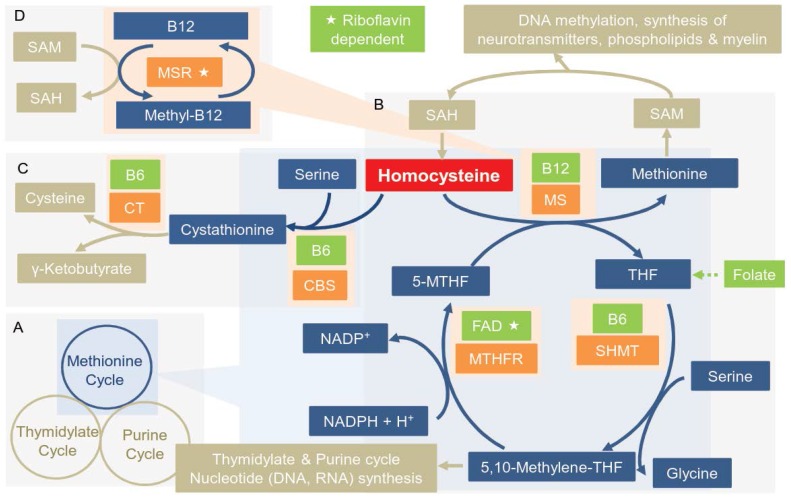
Metabolic pathways of the one-carbon metabolism: (**A**) Overview of the three cycles; (**B**) Methionine cycle: remethylation of homocysteine to methionine; (**C**) Transsulfuartion pathway: Irreversible conversion of homocysteine into cysteine; (**D**) Remethylation of vitamin B12 to its active form; CBS: cystathionine β-synthase; CT: γ-cystathionase; FAD: flavin adenine dinucleotide; MTHF: methylenetetrahydrofolate; MTHFR: methylenetetrahydrofolate reductase; MS: methionine synthase; MSR: methionine synthase reductase; NADP(H): (Hydroxy) Nicotinamide adenine dinucleotide phosphate; SAH: *S*-adenosylhomocysteine; SAM: *S*-adenosylmethionine; SHMT: serine hydroxymethyltransferase THF: tetrahydrofolate.

The OCM is a complex metabolic pathway in which reduced tetrahydrofolate (THF), the active form of folate, acts as co-enzyme in the transfer of methyl groups [43]. It consists of three interrelated cycles, which are the methionine, thymidylate and purine cycles [44] (Figure 1A). Hcy can either be fed into the methionine or the thymidylate cycle (Figure 1A–C): When methionine levels are low, Hcy is remethylated into methionine (Figure 1B,D). For this, a methyl group is transferred from methylenetetrahydrofolate (MTHF) to Hcy by the methionine synthase (MS), resulting in THF and methionine. The latter can be further metabolized into *S*-adenosylmethionine (SAM), which plays a crucial role as a methyl-donor in other metabolic pathways such as DNA methylation or synthesis of neurotransmitters, phospholipids and myelin [44]. 

*S*-adenosylhomocysteine (SAH), remaining after the one-carbon transfer from SAM, is then hydrolyzed back to Hcy [45]. Serine and THF are turned into glycine and 5,10-methylene-THF in a reaction catalyzed by the serine hydroxymethyltransferase (SHMT) [46]. Then, 5,10-methylene-THF is reduced to 5-methylenetetrahydrofolate (MTHF) by the action of the methylenetetrahydrofolate reductase (MTHFR) [47], closing the cycle. If sufficient methionine is available or Hcy is accumulating, Hcy condenses with serine to form cystathionine and subsequently cysteine [45]. This reaction is called the transsulfuration pathway and is mediated by two vitamin B6-dependent enzymes (cystathionine β-synthase (CBS) and γ-cystathionase) [45].

MS depends on methyl-cobalamin, the active form of vitamin B12 [44], as an intermediate methyl carrier, and consequently, adequate amounts of the nutrient are essential to keep the cycle going [48]. Vitamin B12 is regenerated into its active form by the methionine synthase reductase (MSR) through remethylation with one-carbon units from SAM [49,50]. MSR is a flavoprotein [51] and therefore riboflavin dependent. SHMT consists of four subunits and each of those uses pyridoxal-5′-phosphate, the active form of vitamin B6, as a cofactor [52]. MTHFR also uses flavin adenine dinucleotide (FAD), derived from riboflavin, as a cofactor [51]. This highlights the important role B-vitamins play in the OCM and how deficiencies of each of them are likely to disturb its balance in specific ways.

### 3.2. Polymorphisms in Key Enzymes of the OCM

The relationship between B-vitamins, relevant polymorphisms and AD has not been studied in great detail and the potential mechanisms are poorly understood. The association between MTHFR C677T and AD has been studied in most detail, while for the other polymorphism the available evidence is very limited. In addition, a great shortcoming of the majority of studies is that no information on nutritional status in general and on B-vitamins more specifically is provided. We will also review how reduced enzymatic activity due to polymorphisms combined with lack of cofactors caused by inadequate dietary intake might unbalance these metabolic processes, thereby potentially favoring the development of AD.

#### 3.2.1. Methylenetetrahydrofolate Reductase (MTHFR) Polymorphisms

MTHFR is by far the most widely studied enzyme in regard to polymorphisms affecting the OCM and their effect on Hcy levels. While deficiency is relatively rare in humans [53], three common mutations of the MTHFR gene, namely C677T, A1298C, and T1317C, have been proposed for an association with various pathological conditions. However, the T1317C mutation appears to be a silent polymorphism [24]; very limited evidence is available and none of it shows any association with Hcy levels or B-vitamin intakes [54,55], let alone AD and this will therefore not be discussed further in this review.

Globally, the frequency of population homozygote for the MTHFR 677TT mutation is thought to range from close to 0% in Sub-Saharan Africans to 32% in Mexicans [56,57]. Homozygotes for the polymorphism were reported to be more likely to have elevated Hcy levels compared to the population average [58,59,60,61,62,63,64,65,66,67,68] and the mutation constitutes the most frequent cause of moderate hyperhomocysteinemia due to genetic factors [20]. There is some evidence for gender-specific differences: one study found men who were homozygous carriers of the mutation had a much higher risk for significantly elevated Hcy levels than women [61]. In addition, a French study corroborated the above results by showing that genotype affected Hcy levels in men, but not in women [69]. Interestingly, one study found the age-dependent increase in Hcy masked the effect of the mutation and only showed a significant association in the older participants [70]. 

The enzyme activity seems to be reduced by up to 50% [20] due to reduced stability of the association with its cofactor FAD [21,22]. The addition of folate derivatives was shown to stabilize the FAD-MTHFR-folate complex in *Escherichia coli* with the 677TT mutation [22]. In line with this, the effect of the mutation on Hcy levels was more pronounced if folate levels were low [48,54,60,61,63,70,71,72] and it was not apparent in persons with high intakes from supplements (≥400 µg/day) [66]. It was shown that the odds ratio for elevated Hcy levels in this genotype increased from 15 to 175 if plasma folate was ≤3.7 nmol/L [73]. Moreover, hyperhomocysteinemia in persons homozygous for the 677T mutation could be reversed or reduced with folic acid supplementation [47,73], while this had no effect in persons carrying the wild type allele [47]. A folate depletion–repletion study in elderly women showed a more pronounced decrease in serum folate levels accompanied by a steeper increase of Hcy levels in homozygous carriers of the mutation compared to the wild type [74]. After repletion, both serum folate and Hcy levels normalized to levels comparable with those of the participants with the wild type [74]. All this indicates that individuals with the MTHFR 677TT genotype may have higher folate requirements and might benefit even more from supplementation [71] as increasing folate intakes could compensate for the reduced activity of the MTHFR. Multivitamin supplements showed a positive impact on levels of other B-vitamins such as vitamin B12 and pyridoxal 5′-phosphate [47] and might therefore also beneficially affect Hcy levels. Riboflavin status was also negatively associated with Hcy levels in carriers of at least one copy of this polymorphism [75]. In particular, Hcy levels were increased in persons homozygous for 677TT with marginal or low riboflavin status compared to heterozygous and wild types, which was not the case if the vitamin status was adequate [72]. In line with this, daily supplementation with 1.6 mg improved riboflavin status in all subjects with low levels at baseline, but Hcy levels only decreased significantly (by 40%) in subjects who were homozygous carriers of the mutation [76]. Moreover, the impact of riboflavin status on Hcy levels was more important in homozygous carriers of the 677TT mutation with low folate status [77]. Consequently, these data indicate that both riboflavin and folate can independently compensate the decreased MTHFR enzymatic activity due to the mutation.

While the effect of vitamin B6 on Hcy was found to be inconsistent, Hustad and colleagues [78] suggest that the effect is particularly evident in persons homozygous for the MTHFR 677T mutation and that interactions with other B vitamins might further complicate the relationship. Hcy levels were found to be inversely associated with vitamin B6 status if riboflavin levels were adequate, but plasma folate levels were low [72]. If re-methylation of Hcy via the methionine cycle (Figure 1B) is not possible due to lack of folate, the alternative pathway for Hcy is condensation with serine to cystathionine and this is catalyzed by the vitamin B6-dependent CBS (Figure 1C). However, if this pathway is also disturbed due to insufficient levels of vitamin B6, Hcy seems to accumulate. The role of vitamin B12 in persons with 677TT genotype is not completely clear: Vitamin B12 levels did not seem to have any effect on the risk of hyperhomocysteinemia in the 677TT genotype in some studies [60,71], while others reported a negative association between Hcy and serum vitamin B12 levels, particularly in person homozygous for 677T [54,79]. Moreover, Hcy levels were found to be higher in homozygous carriers of the mutation with low vitamin B12 levels, particularly if they did not take folate supplements [66]. The mechanism for such an effect is unknown, but it has been suggested it might be due to a coexisting mutation within the OCM [48]. 

The A1298C mutation in MTHFR gene was reported in approximately 10% of Canadians [24]. The prevalence seems to differ between ethnic groups: while non-Hispanic whites in the U.S. showed a similar prevalence of homozygous carriers (~12%) as that reported for Canadians, in Mexican Americans, the prevalence was ~20% and in non-Hispanic blacks it was just over 1% [66]. In itself, the A1298C mutation was not associated with elevated Hcy levels in either heterozygotes or homozygotes in most studies [54,61,62,63,66,70,80,81] and the reduction in MTHFR activity is lower than for the 677TTTT mutation (~70% of wild type) [23]. However, in vitro studies indicate a synergistic effect for the two mutations [23], Hcy levels were found to be highest [61,82] and the corresponding red cell folate level lowest in individuals with both mutations [61]. No significant effects were found for the combinations of genotypes and serum folate or vitamin B12, but this might be due to the low prevalence of the recombinant genotype [61,82]. In other studies, the activity of MTHFR was further reduced than what would have been expected from C677T alone in individuals heterozygous for C677T and A1298C (none of the subjects was homozygous for both mutations), which was accompanied by increased Hcy and decreased plasma folate [23,24,83]. Carriers of the wild type for both polymorphisms on the other hand were found to have the lowest Hcy levels compared with other combinations of the genotypes [84]. Again, the effect seems to be more pronounced in individuals with low folate levels [23].

In addition, plasma vitamin B6 levels were lower in individuals heterozygous for C677T carrying at least one copy of the mutation for A1298C compared to those who were homozygous for A1298C, but this might have been due to differences in supplement use between the groups [23]. Moreover, in doubly heterozygous subjects, plasma vitamin B12 was a significant predictor of Hcy levels, which was not the case for those who were wild type for at least one of the polymorphisms [48]. The authors conclude that these people would benefit from an increase in vitamin B12 status, as this would help reducing or normalizing Hcy levels [48]. 

#### 3.2.2. Methionine Synthase (MS) Polymorphism

Several polymorphisms in MS have been identified, which might potentially be relevant for the Hcy metabolism [25,26]. The most prevalent is the A2756G polymorphism with an allele frequency of around 20% [25,26,85,86,87]. A number of studies in both healthy and sick individuals of different age and gender groups assessed its effect on Hcy or B-vitamin levels and found no or only marginal effects that failed to reach statistical significance [61,86,87,88,89,90,91]. Given the relatively low prevalence of homozygous mutation in the gene encoding for MS, larger studies might be able to shed more light on the relationship between the different genotypes, levels of B-vitamins and Hcy concentrations. One study in more than 1200 healthy men between the ages of 50 and 61 years found that carriers homozygous for the more common AA genotype had higher Hcy levels than those with at least one copy of the G mutation, independent of folate or vitamin B12 status [60]. Similarly, fasting and post-methionine load Hcy levels were lower in individuals with at least one copy of the 2756G allele [59]. Moreover, an additive effect on Hcy levels was reported in carriers who have at least one copy of the MTHFR 677T allele and who are homozygous for MS 2756A [60]. 

In addition, Ma and colleagues report a trend towards a protective effect of the GG genotype for colorectal cancer despite the lack of association with Hcy, indicating an effect via a different mechanism, possibly via DNA methylation [91]. In line with this, the AG genotype was associated with increased erythrocyte folate and lower risk for myocardial infarction, but not Hcy or vitamin B12 levels, compared to the wild type (only one patient was homozygous for the mutation and was therefore not included in the analysis) [92]. Reduced activity of MS and the resulting decrease in SAM could affect DNA methylation and/or synthesis of neurotransmitters, phospholipids and myelin (Figure 1B), which in turn could contribute to the development of the AD. Hcy levels could still be kept in the normal range by condensing it with serine to cystathionine (Figure 1C). This is in line with the finding that the mutation correlated with cystathionine levels [70], indicating a preference for transsulfuration rather than re-methylation in the 2756AG/GG genotype. In line with this, one study found moderately increased Hcy levels in persons with the AA genotype, which increased with decreasing levels of vitamin B6, but seemed independent of folate and vitamin B12 status [93]. 

Moreover, a study in American men aged ~40 to 80 years found that Hcy levels decreased with increasing number of copies of the 2756G allele in healthy controls, but not in cases with a history of myocardial infarction [94]. It has been proposed that in conditions of elevated oxidative stress, functional vitamin B12 deficiency arises as the recycling into its active form cannot keep up with the rate of its oxidation [95]. Consequently, it can be speculated that the effect of the polymorphism on enzyme activity in the above-mentioned patients is masked by the stronger effect of vitamin B12 oxidation. Whether this underlying mechanism is relevant for the etiology of AD needs to be established. However, it is conceivable to assume such a link given the elevated levels of oxidative stress found in AD patients’ brains.

#### 3.2.3. Methionine Synthase Reductase (MSR) Polymorphism

Another relatively common mutation affecting an enzyme of the OCM is the A66G mutation in the gene encoding for the MSR. In a range of studies, it was reported that ~25% to 30% of Caucasians were homozygous carriers of the mutation [61,72,96]. Data from case-control studies indicate a great range between countries (for a review see [97]), but also between ethnic groups within one country: In the U.S. ~30% non-Hispanic whites were found to be homozygous for the mutation compared to ~20% Ashkenazi Jews, ~8% to 10% non-Hispanic blacks and ~7% Mexican Americans [66,98], while in Muslims in India, ~50% were carriers of two copies of the mutation [97]. 

It was reported that the mutation lead to a less efficient regeneration of vitamin B12 [27] and it has been proposed as a risk factor for elevated Hcy levels [51] as it reduces its conversion into methionine. Another consequence of this impairment of the OCM is the reduced availability of SAM for DNA methylation [97]. However, the majority of studies does not confirm an effect on Hcy levels [66,70,82,88,96,99,100,101] and only two studies found significantly [49,102] and borderline significantly higher Hcy [61] levels in homozygous carriers of the mutation.

Given the role of MSR in recycling vitamin B12 and thereby contributing to the remethylation of Hcy, the decreased activity caused by the mutation can be assumed to be particularly critical if vitamin B12 levels are low. This was confirmed by a study showing that in persons with low plasma cobalamin levels (≤273 pmol/L), Hcy levels were higher in carriers of the mutant allele, if their riboflavin status was adequate [72]. In other words, adequate vitamin B12 levels seem to be able to compensate the reduced enzymatic activity, and the impairment due to inadequate riboflavin levels masks that due to genetic variation. If Hcy cannot be turned into methionine, the transsulfuration pathway involving the vitamin B6-dependent CBS will be activated to regulate its levels (Figure 1C). It is therefore not surprising that vitamin B6 status has an impact on the effect of the A66G polymorphism on Hcy levels [72]. 

There might also be an interaction between the different genotypes: in non-Hispanic whites homozygous for MTHFR 677T, there was a significant trend towards lower Hcy levels with increasing numbers of copies of 66G, which was not the case for the 677CC or CT genotypes [66]. While the MSR genotype in itself had no effect on Hcy in a study in healthy women, there seems to be an effect in combination with the MTHFR 677TT genotype [82]. However, the authors conclude that due to the small sample size in the MTHFR 677TT/MSR 66AA and GG groups, they failed to detect potential differences in plasma Hcy between these groups.

Further research is needed to assess the effect of this mutation, particularly in combination with other polymorphisms affecting the OCM and/or in individuals with inadequate status of one or more of the relevant B-vitamins. Brown and colleagues [100] showed an effect of the mutation on the risk for coronary artery disease, but not on Hcy levels, indicating that a mechanism other than elevated Hcy levels might be relevant. Given that vascular diseases seem to increase the risk of developing AD [103], this link should be further investigated.

Less is known about the C524T mutation in the gene encoding for MSR, for which ~14% homozygous carriers were found in a group of healthy Spaniards, while nearly 60% had at least one copy of the mutation [72]. It seems to affect the enzyme structure in the region between the binding domains for flavin mononucleotide and FAD/(Hydroxy) Nicotinamide adenine dinucleotide phosphate (NADPH), respectively [27]. As for the A66G variant, this mutation reduced the efficacy of B12 regeneration by MSR [27]. In carriers of the C524T mutation, Hcy was significantly higher than in controls if vitamin B12 levels were low, while riboflavin status had no clear effect [72]. For both mutations of the MSR, vitamin B6 levels were inversely associated with Hcy levels in persons with optimal riboflavin and vitamin B12 levels [72], highlighting again the importance of the transsulfuration pathway for keeping Hcy in the normal range when re-methylation is impaired. 

#### 3.2.4. Cystathionine β-Synthase (CBS) Polymorphism

Relatively rare mutations of the gene encoding for CBS are frequently found in patients with homocystinuria, but they do not seem to be more common in persons with moderately elevated plasma Hcy levels and were consequently considered to be of minor importance as risk factors for the general population [90,104,105,106,107]. In addition, some rare mutations in the gene encoding for CBS have been reported to have beneficial, albeit statistically not significant effects on Hcy levels. These mutations, however, were not regarded as significant due to their low prevalence [108]. However, a more common 68 base pair (bp) insertion in exon 8 in the gene encoding for CBS [28] might be relevant. The prevalence of this insertion in healthy men and women from Northern Ireland was around 18% [61] and around 12% in healthy US controls [109]. In the US, allele frequency has been reported to be significantly higher in non-Hispanic blacks (~26%) compared to non-Hispanic whites (~8%) or Mexican American individuals (~6%) [66]. It had been proposed that insertion had no effect on the enzyme activity [109] and assessed on its own, its effect on Hcy is inconsistent: while some found no effect on Hcy [61,70], others showed a trend towards lower levels at least in specific subpopulations [59,60,66,89,110].

A few studies that assessed the effect of combined polymorphisms found that the insertion is capable of compensating the negative effect of MTHFR 677TT and MS 2756 AA [59,60,108]. However, in another study, a combination of homozygous 677T and 68 bd led to a further increase in Hcy levels, albeit in a very small sample (*n* = 4) [70]. While in black South Africans, the insertion itself had no effect on Hcy, in combination with MTHFR 677TT, those without the insertion had the highest Hcy levels [29]. Similarly, a different mutation of CBS (9276 GA genotype compared to 9276 GG, no 9276 AA in the study) led to an increase in Hcy levels in individuals homozygous for the MTHFR 677T mutation compared to other genotypes [29]. Moreover, increasing numbers of repeat units of the 31-bp *variable number of tandem repeats* polymorphism in the non-coding sequence of CBS at the boundary of exon 13 to intron 13 were found to decrease CBS activity and increase Hcy levels [30]. Frequency and position of these seem to vary between different ethnic groups [111]. Albeit inconsistent, these results highlight the importance of the transsulfuration pathway as an alternative to catabolize Hcy if remethylation is impaired. In line with this, the protective effect of the insertion on Hcy appears to be independent of folate and vitamin B12 status [60]. 

#### 3.2.5. Serine Hydroxymethyltransferase (SHMT) Polymorphism

For SHMT, a polymorphism has been described at the position C1420T. While it has not been studied extensively, one study reports that women with the 1420CC genotype had significantly increased Hcy and decreased red cell and plasma folate levels [31]. In another study in patients with coronary artery disease, the mutation was also associated with lower levels of Hcy, higher plasma folate concentrations and decreased markers of oxidative stress [112].

## 4. Proposed Mechanisms Linking Polymorphisms, Hcy, B-Vitamins and AD

The data presented on polymorphisms in the genes encoding for key enzymes in the OCM and their interaction with various B-vitamins highlights the complex relationship between the various steps of these metabolic pathways. Genetic factors affecting the OCM alone are likely to play a relatively minor role in the overall risk of developing AD: 9% of variation in Hcy levels could be explained by differences in the polymorphisms for MTHFR, MS, MTR and CBS, while folate and vitamin B12 status are thought to be responsible for 35% of the variance [61]. Combining these genetic and nutritional factors increased the effect to 42%in relatively young subjects (20–25 years of age) [61]. The authors chose this age group as they expected the genetic effects to be less masked by a range of environmental influences that accumulate over a lifetime [61]. They suggest that more subtle genetic effects might only manifest in combination with longer-term exposure to other factors such as smoking [61]. In any case, polymorphisms help to understand the complexity of the metabolic system and explain some of the inconsistencies encountered in studies trying to link nutritional factors with risks for diseases.

**Figure 2:**
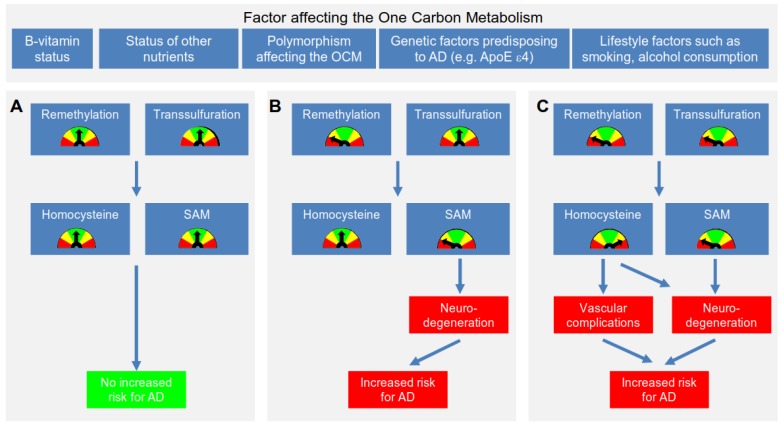
Proposed framework for the effect on genetic, nutritional and lifestyle factors on the development of Alzheimer’s disease: (**A**) Balance between remethylation and transsulfuration results in adequate levels of homocysteine, DNA synthesis, repair and methylation as well as synthesis of neurotransmitter, phospholipids and myelin and consequently no increase in the risk of Alzheimer’s disease; (**B**) Remethylation is decreased, while homocysteine is still kept in the normal range via transsulfuration, resulting in reduced DNA synthesis, repair and methylation as well as synthesis of neurotransmitter, phospholipids and myelin and consequently, an increase in the risk of Alzheimer’s, but not vascular disease; (**C**) Remethylation and transsulfuration are decreased, resulting in reduced DNA synthesis, repair and methylation as well as synthesis of neurotransmitter, phospholipids and myelin and consequently, an increase in the risk of Alzheimer’s, also due to compromised vascular health; AD: Alzheimer’s disease; ApoE: Apolipoprotein E; OCM: One-carbon metabolism; SAM: *S*-adenosylmethionine.

The evidence presented above shows that lack of substrate or reduced enzymatic activity in one step in the OCM can be compensated at least partially or results in a shift to a different pathway. Consequently, it seems that health is only affected if these copying mechanisms fail due to a combination of more than one polymorphisms and/or inadequate supply of relevant vitamins (Figure 2). It is therefore not surprising that studies concentrating on a single polymorphism and its association with AD failed to show a consistent picture: A number of studies did not detect a difference in the frequency of the MTHFR C677T genotype in AD patients and controls [113,114,115,116,117,118,119,120,121,122,123,124,125,126], which is probably only partially due to the small sample sizes. Other studies and meta-analyses found an effect of the C677T polymorphism on AD [127,128,129], but there seemed to be some differences between the ethnicities [127,130]. Unfortunately, no study actually took into account the different polymorphisms of key enzymes in the OCM in combination with B-vitamin status. Importantly, one study showed that despite a lack of difference in C677T genotypes or Hcy levels between patients and controls, plasma Hcy concentrations were significantly higher in patients with dementia who were either TT or CT and had low folate levels (<5.7 nmol/L) compared to those with adequate folate levels or CC genotype [131]. 

AD is a multifactorial disease, which is poorly understood and a range of hypothesis have been proposed for its etiology, which are reviewed in detail elsewhere [9,132]. According to the authors of a recent review, low folate and vitamin B12 status contribute to the development of cognitive impairment directly and via elevated Hcy levels [16]. These mechanisms will be discussed in the following sections.

### 4.1. Proposed Mechanism Linking Hcy & AD

A range of mechanisms have been proposed for the link between elevated Hcy and AD and preclinical studies show that hyperhomocysteinemia, induced by genetic manipulation or by B-vitamin deficiency, causes known hallmarks of AD such as accumulation of amyloid-β peptide [133,134,135,136] and intensified tau protein hyperphosphorylation in the brain [137]. An autopsy study showed a clear association between Hcy levels and neurofibrillary tangles, a known hallmark of AD, with an odds ratio of having such deposits of 2.60 (95% confidence interval 1.28–5.28) when comparing the top with the bottom Hcy quartile [138]. A prospective study showed greater brain atrophy in AD patients with higher Hcy levels [116] and this association between Hcy and grey matter atrophy has been confirmed by a range of studies (See review by Smith and Refsum [16]).

Amyloid plaque formation is thought to be an important event in the etiology of AD [139,140] and there is evidence that elevated levels of Hcy can impact the plaque formation by reducing the clearing rate of amyloid-β in the brain of mice [141]. Moreover, amyloid-β levels increased in rats after injection of Hcy into their brain and this was accompanied by loss in spatial memory [142]. Folate and vitamin B12 supplementation was able to lessen these effects [142]. A further piece of the puzzle is the finding that Hcy can bind to amyloid-β in vivo and in vitro, thereby triggering the formation of interconnections and subsequently aggregates [143]. Moreover, these deposits can induce oxidative stress, another important element in the etiology of AD [144,145]. 

The effect of elevated Hcy on brain capillaries is a further mechanism through which an impaired OCM might facilitate the development of AD [146] (Figure 2C). It has been postulated that elevated Hcy levels due to genetics or dietary inadequacies may compromise vascular health, thereby contributing to dementia and AD [131]. Hcy is thought to affect endothelial integrity by promoting the generation of peroxides, but also by reducing the availability of nitric oxide through a reduction of intracellular glutathione peroxide levels [147]. Moreover, while these vascular effects might be more prominent in individuals who are not otherwise genetically predisposed to AD, it has been speculated that there might be a more direct effect on brain cells in those with the ApoE ε4 genotype [146]. 

Increased levels of Hcy were shown to be a risk factor for shrinkage of specific brain regions including the bilateral hippocampus and parahippocampal gyrus, retrosplenial precuneus, lingual and fusiform gyrus, which is a key component of the AD process and is associated with cognitive decline [40]. In rats, it has been shown that exposure to Hcy leads to apoptosis in hippocampal neurons by inducing a cascade that results in DNA damage, decline of mitochondrial membrane potential and eventually nuclear disintegration, possibly triggered by nicotinamide adenine dinucleotide and adenosine triphosphate depletion [148]. Hcy was shown to accumulate in neurons as it is rapidly taken up via specific membrane transporters [149]. These changes might then increase the vulnerability of neuronal cells to oxidative stress and further contribute to the development of AD [148]. 

Evidence from animal studies also indicates that Hcy is likely to contribute to cognitive decline, but also that its levels further increase as a result of neurodegeneration [150]. The authors conclude that dietary intake or supplementation with B-vitamins might be able to break this vicious cycle. Moreover, in many conditions that are related to oxidative stress, including neurodegenerative diseases, a simultaneous elevation of Hcy and reduced level of B-vitamins, particularly folate, has been reported [151]. It has consequently been proposed that folate requirements might be increased due to irreversible oxidation and that hyperhomocysteinemia might be a consequence of the pro-oxidative environment and not just a result of inadequate intakes [151,152]. 

### 4.2. Further Mechanisms Linking an Impaired OCM to the Development of AD

As the framework in Figure 2 shows, Hcy levels in the normal range do not necessarily mean that there is no disturbance of the OCM. Mechanisms such as DNA repair can be reduced due to specific polymorphisms alone or in combination with low levels of folate, riboflavin and/or vitamin B12, while Hcy is catabolized via transsulfuration. This pathway has been known to be upregulated if methionine recycling is reduced in order to keep Hcy levels low [153]. However, as the switch affects the substrates or methyl donors for essential pathways, such imbalances not only compromise DNA synthesis, repair and methylation, but also the availability of neurotransmitters, phospholipids and myelin (See below). Hcy can further be re-methylated to methionine via the betaine pathway in the liver or kidney [154], but not in the brain [155]. The balance between these pathways depends on an elaborate feedback loop, but also on the availability of nutrients such as folate, vitamin B6, B12 and methionine as well as the methyl-donors choline and betaine [154]. Interestingly, one study found that choline was a strong positive predictor of Hcy levels in Mexican American men with the MTHFR 677TT, but not the 677CC, genotype who had low folate levels [156].

It has also been shown that decreased activity in one enzyme of the OCM can trigger downregulation in the gene expression for key enzymes in alternative pathways, thereby affecting the balance, e.g., between DNA methylation and synthesis [157]. In addition, there is evidence that during folate deficiency, mechanisms are in place to preserve thymidylate and consequently DNA synthesis at the expense of Hcy remethylation [158]. Even though one has to be careful to draw causative conclusions from associations, it is conceivable that such imbalances contribute to the development of AD as SAM levels in postmortem brains of AD patients were reduced compared to non-demented controls [159] and changed methylation patterns were found in postmortem analysis of specific brain regions of AD patients [160]. 

Van Driel and colleagues argue that the ratio of SAM to SAH might be a more relevant predictor of health outcomes due to impaired OCM [161] and this might also apply in the case of AD. SAM plays a crucial role as methyl-donor in other metabolic pathways such as DNA methylation or synthesis of neurotransmitters, phospholipids and myelin [44] (See Figure 1B). In the brain, SAM-dependent methylations are of particular importance [162,163,164,165] and a lack seems to favor the accumulation of amyloid precursor protein and phosphorylated tau protein, validated hallmarks of AD [133,137,166,167,168]. SAM is the major methyl-group donor for DNA methylation; it is involved in the regulation of enzymes necessary for these processes, such as the DNA methyltransferase, and inadequate availability of SAM is thought to play a role in the development of neurodegenerative diseases such as AD (For review see Fuso 2013 [169]). Evidence from transgenic mouse model of amyloid deposition shows that folate deficiency decreased SAM levels and DNA methyltransferase activity in the hippocampus and consequently increased activity of genes thought to be involved in the formation of amyloid plaque [170]. 

Polyunsaturated fatty acids, docosahexaenoic acid (DHA) in particular, play an important role in cognitive health as they are implicated in synaptic functions and signaling pathways, but also for the structure of membranes in the brain and imbalances are thought to be implicated in a range of neuropsychiatric diseases including AD (See review by Liu and colleagues [171]). A crucial step to ensure adequate supply with essential fatty acids to tissues such as the brain includes the methylation of phosphatidylethanolamine to phosphatidylcholine, which requires the phosphatidylethanolamine methyltransferase (PEMT) [172]. PEMT is thought to be regulated by SAM and SAH concentrations [173] and an impaired OCM can therefore be expected to limit the availability of essential fatty acids such as DHA to the brain. This is in line with the findings of a study that found significantly decreased DHA mobilization from the liver likely due to elevated levels of Hcy and SAH AD patients compared to healthy controls [18]. Moreover, DHA levels in the brains of AD patients were lower than in those of controls and at least in some regions of the brain (temporal and mix-frontal cortex, but not cerebellum) they were negatively correlated with the degree of cognitive decline [174].

It has been postulated that early on in the disease, oxidative stress levels increase due to mechanisms most likely unrelated to the OCM [95]. As a consequence, functional vitamin B12 deficiency can develop if the rate of oxidation surpasses its recycling [95]. This effect is likely more pronounced if the activity of MSR is reduced due to a polymorphism. Elevated Hcy levels would therefore be a consequence of changes occurring due to the AD pathophysiology, but they might then also contribute to its progression [95]. The MSR A66G was found to be correlated not only with Hcy levels, but also with markers of oxidative stress [112]. It has been suggested that the reduction in MS activity due to lack of vitamin B12 might mask the more subtle decrease in activity due to an MS polymorphism, thereby further complicating the association between the genotype and AD [175].

Dorszewska and colleagues [176] report an increase of markers for oxidative stress as well as Hcy levels in AD patients, while in elderly controls, there was an age-related, but less pronounced increase in the latter, but not the former. Moreover, even though ApoE ε4 itself does not seem to be linked to elevated Hcy levels [115], the increased level of oxidative damage thought to be linked to the ApoE ε4 genotype might be aggravated if the OCM is disturbed. Studies in mice demonstrate that folate might play an important role in countering the effect of elevated oxidative stress prevalent in brains of ApoE ε4carriers [177,178]. Markers of oxidative stress in the central nervous system of ApoE knockout mice only increased following an iron challenge if folate was deficient [178]. Folate deficiency was associated with increased Hcy and a reduced ability to counter oxidative stress as it was shown to decrease the activity of key antioxidant enzymes, namely the Cu-Zn superoxide dismutase and the glutathione peroxidase [179]. While it was long assumed that the oxidative pentose phosphate pathway was the main source of NADPH, it was recently shown that the OCM is equally important in providing this important reducing agent [180]. An impaired OCM can therefore be expected to increase the vulnerability to oxidative stress by decreasing the cell’s oxidative defense mechanisms. Wakutani and colleagues therefore propose that an impaired folate metabolism due to the MTHFR polymorphism or inadequate dietary supply might enhance the adverse effect of ApoE ε4 on the etiology of AD [181]. 

In addition, studies in mice that are not genetically predisposed to AD show that inadequate supply with B vitamins in itself can cause cognitive decline [146]. One potential mechanism is that impaired DNA repair due to deficiency of folate seems to increase oxidative neuronal damage induced by amyloid beta-peptide [182]. It is postulated that damage to mitochondrial DNA accumulating with age leads to increased oxidative stress, which—in the absence of efficient repair mechanisms—causes neurodegeneration (for a review see Swerdlow et al. 2014 [17]). The activity of CBS is thought to increase in response to oxidative stress [183], which might result in a further imbalance of the OCM that could potentially contribute to the development of the disease.

Age itself might further contribute to impaired OCM as there was an age-dependent decrease in THF accompanied by an increase in products of its oxidation, which are biologically inactive [184]. Hcy levels were elevated in both dementia patients and elderly controls when compared to a younger group of neurological patients without dementia [131]. Similarly, an association between age and Hcy levels as well as a negative association between the former and serum folate and vitamin B12 concentrations were found in the combined as well as stratified analysis of AD patients and controls [10]. In addition to the aging process, lifestyle factors such as alcohol consumption and smoking might also influence the interaction between B-vitamins, polymorphisms of the OCM and AD [60,91]. A vicious circle between impaired OCM and oxidative stress seems to develop in the elderly, particularly in certain genotypes prone to impaired cognitive health. It was suggest that carriers of the 677T mutation might still be able to compensate the imbalance in the folate metabolism under normal circumstances, but if vitamin B12 supply is also inadequate, an effective compensation might no longer be possible [185]. This ties in with the findings of another study that reported a weak, but significant association between the MSR 66G mutation and the risk for AD as well as with the severity of the disease, particularly in combination with the ApoEε4 genotype [123]. 

Many questions remain concerning the proposed mechanisms and given their key roles in a range of processes pertinent to brain health, it is likely that more than one is relevant for the etiology of AD. 

## 5. Dietary Intake of B-Vitamins

Current intake recommendations do not take into account potentially increased needs due to reduced enzymatic activity caused by a polymorphism, as the mechanisms are not understood well enough to adapt them accordingly. Until this is possible, it is advisable to assure intakes of B-vitamins in all age groups are in line with the available recommendations to reduce the risk of the developments that eventually result in AD. 

Worryingly, for part of the general population even in affluent countries, this is not the case. A relatively recent analysis of data from the National Health and Nutrition Examination Survey reported elevated Hcy levels in ~6% of the U.S. population aged ≥19 years, with levels ranging from >3% in 19–39 year-olds to ~18% in those ≥60 years old [186]. Despite mandatory folic acid fortification, around 5% and 15% of men and women, respectively, in the age range of ≥19years have folate intakes below the Estimated Average Requirement (EAR) in the United States [187]. Similar figures were given by Agarwal and colleagues [188], who also report intakes of vitamin B6 to be low for ~15%. In different European countries, intakes below EAR range from 0% to 40% for vitamin B12 and from 10% to just over 90% for folate in adults aged 19–64 years [189]. In Ireland, voluntary fortification as well as dietary supplements significantly contributed to achieving adequate folate intakes, but still, nearly 70% of women aged 18–50 years had suboptimal serum folate levels [190]. Vitamin B12 deficiency is typically seen as a problem of the elderly due to malabsorption [191,192,193,194]. However, Bailey and colleagues argue that even if the proportions of people with deficiencies in the general population are not very high, the absolute number of affected persons is still significant [186]. 

As is often the case in nutrition, B-vitamins can only function properly if the supply of other essential nutrients is assured: It has been shown that an intervention with B-vitamins in elderly with mild cognitive impairment only showed beneficial effects if their omega-3 fatty acid status, particularly DHA, was adequate [195]. Worryingly, it has been shown that intakes of DHA are low in many regions of the world [196]. Other nutritional inadequacies likely also play a role in the development of AD, which further highlights the importance of a diet that supplies all essential nutrients in adequate amounts through.

## 6. Conclusions

The evidence presented shows that persons with specific genotypes are more susceptible to imbalances in the OCM, resulting in increased levels of Hcy, insufficient DNA repair, methylation and/or synthesis as well as reduced availability of neurotransmitters, phospholipids and myelin. This can facilitate the development of AD via a range of—as of yet—poorly understood mechanisms, particularly, but not exclusively, if other risk factors such as the ApoE ε4 polymorphism predispose an individual to the disease. The reduced enzymatic activity can be compensated at least to some degree by adequate intakes of the relevant B-vitamins. Even though supplementation with folate, vitamin B6 and B12 might be able to slow the progression of dementia at an early stage [37,39,40], by the time overt clinical signs appear it might be too late to reverse the decline [197]. This emphasizes the importance of a life-long adequate intake of B-vitamins for prevention of cognitive decline and dementia.

The relationship between polymorphisms of the OCM, intakes of B-vitamins and AD can only be resolved with well-designed long-term cohort studies with detailed neuropsychological and vascular measurements. Given the long latency period between the occurrence of elevated Hcy as well as oxidative stress levels and the first symptoms of cognitive decline, studies should be initiated with healthy, middle-aged subjects. Moreover, these indicators have to be assessed at regular intervals to allow for a more in-depth understanding of the mechanisms eventually leading to AD. Until these issues are resolved, efforts should be made to ensure adequate intakes of all B-vitamins via the diet, fortified foods and possibly dietary supplements. 

## Figures and Tables

**Table 1:** Polymorphisms relating to key enzymes in the one-carbon metabolism that are potentially relevant to the development of Alzheimer’s disease (AD).

Enzyme	Polymorphism	Reference
MTHFR	C677T	Schwahn and Rozen 2001 [20], Yamada et al., 2001 [21], Guenther et al., 1999 [22]
	A1298C	Weisberg et al., 2001 [23]
	T1317C	Weisberg et al., 1998 [24]
MS	A2756G	Leclerc et al., 1996 [25], Chen et al., 1997 [26]
MSR	A66G	Olteanu et al., 2002 [27]
	C524T	Olteanu et al., 2002 [27]
CBS	68 bp insertion at exon 8	Sebastio et al., 1995 [28]
	G9276A	Nienaber-Rousseau et al., 2013 [29]
	31 bp variable number of tandem repeats	Lievers et al., 2001 [30]
SHMT	C1420T	Heil et al., 2001 [31]

bp: base pairs; CBS: Cystathionine β-synthase; MSR: Methionine synthase reductase; MS: Methionine synthase; MTHFR: Methylenetetrahydrofolate reductase; SHMT: Serine hydroxymethyltransferase.
